# Differences in immunoreactive trypsin values between type of feeding and ethnicity in neonatal cystic fibrosis screening: a cross-sectional study

**DOI:** 10.1186/s13023-014-0166-9

**Published:** 2014-11-07

**Authors:** Ernesto Cortés, Ana María Roldán, Antonio Palazón-Bru, María Mercedes Rizo-Baeza, Herminia Manero, Vicente Francisco Gil-Guillén

**Affiliations:** Pharmacology, Paediatrics and Organic Chemistry Department, Miguel Hernández University, San Juan de Alicante, Spain; Clinical Analysis Department, Alicante General University Hospital, Alicante, Spain; Clinical Medicine Department, Miguel Hernández University, Carretera de Valencia-Alicante S/N, 03550 San Juan de Alicante, Spain; Nursing Department, University of Alicante, San Vicente del Raspeig, Spain

**Keywords:** Cystic fibrosis, Neonatal screening, Nutrition, Ethnic groups

## Abstract

**Background:**

We studied the differences in immunoreactive trypsin (IRT) in neonatal screening for cystic fibrosis (CF) associated individually with the age of the newborn, ethnicity and environmental temperature. In this study, we determine the overall influence of environmental temperature at birth, gender, feeding, gestational age, maternal age and ethnic origin on an abnormal IRT result.

**Methods:**

Cross-sectional observational study. A sample was selected of newborns from Alicante (Spain) who underwent neonatal CF screening in 2012–2013. Primary variable: abnormal IRT levels (≥65 ng/ml). Secondary variables: gender, maternal origin, maternal age (years) (<20, 20–40, >40), gestational age (weeks) (<32, 32–37, >37), type of feeding (natural, formula, mixed and special nutrition), >20 days from birth to blood collection, and average temperature during the month of birth (in°C). Using a multivariate logistic regression model the adjusted odds ratios (ORs) were estimated to analyze the association between atypical IRT levels and the study variables. The α error was 5% and confidence intervals (CI) were calculated for the most relevant parameters.

**Results:**

Of a total of 13,310 samples, 199 were abnormal (1.34%). Significant associated factors: feeding method (natural → OR = 1; mixed → OR = 0.53, 95% CI: 0.31-0.89; formula → OR = 0.72, 95% CI: 0.48-1.07; special → OR = 21.88, 95% CI: 6.92-69.14; p < 0.001).

**Conclusions:**

Newborns receiving special nutrition have a 20-fold higher risk for abnormal IRT levels, and screening is advisable once normalized feeding is initiated. It is advisable to consider ethnic variability. Seasonality was not important.

## Spanish abstract

**Antecedentes:**

Se ha estudiado la diferencia de tripsina inmunorreactiva (TIR) en el cribado neonatal de fibrosis quística (FQ) relacionados individualmente con edad del recién nacido, etnia y temperatura ambiente. En este estudio nosotros determinamos la influencia global de temperatura ambiente al nacimiento, sexo, alimentación, edad gestacional, edad de la madre y origen étnico sobre la TIR alterada.

**Métodos:**

Estudio observacional transversal. Se seleccionó una muestra de recién nacidos de Alicante (España) a los que se les realizó cribado neonatal de FQ en 2012–2013. Variable principal: niveles alterados de TIR (≥65 ng/ml). Variables secundarias: sexo, origen de la madre, edad materna (años) (<20; 20–40; >40), semanas de gestación (<32; 32–37; >37), tipo de lactancia (natural, artificial, mixta y nutrición especial), >20 días desde el nacimiento a la extracción de sangre y temperatura media del mes de nacimiento (en°C). Con un modelo multivariante de regresión logística se estimaron las odds ratio ajustadas (ORs) para analizar la relación entre la TIR atípica y las variables del estudio. El error α fue del 5% y se calcularon intervalos de confianza (IC) para los parámetros más relevantes.

**Resultados:**

De un total de 13,310 muestras, 199 estuvieron alteradas (1.34%). Factores asociados significativos: tipo alimentación (Materna → OR = 1; Mixta → OR = 0.53, IC 95%: 0.31-0.89; Fórmula → OR = 0.72, IC 95%: 0.48-1.07; Especial → OR = 21.88, IC 95%: 6.92-69.14; p < 0.001).

**Conclusiones:**

Los recién nacidos con nutrición especial tienen 20 veces más riesgo de TIR alterada, siendo aconsejable cribarlos una vez inicien alimentación normalizada. Es aconsejable tener presente la variabilidad étnica. La estacionalidad no fue importante.

## Background

Cystic fibrosis (CF) is one of the rare diseases for which newborn screening is recommended [1] based on the determination, in the first week of life, of immunoreactive trypsin (IRT) in dried blood spots collected on filter paper, because of its increased level in CF patients, likely related to obstruction of the pancreatic ducts [2].

Studies have shown that children diagnosed through screening have better nutritional and respiratory parameters [3,4], better intellectual development [5,6], and increased survival [7] than those diagnosed after presenting clinical manifestations [8-11].

These benefits, coupled with successful experiences in other countries and communities, have allowed implementation of newborn screening since that time. Newborn screening in the Valencian Community began in 2012 by quantification of IRT, which is elevated in the blood samples of newborns with cystic fibrosis due to pancreatic duct obstruction with trypsin reflux into the blood. The critical point is the establishment of the cutoff in the first sample. Each laboratory should establish its own cutoffs, considering that the IRT concentration is dependent on the age of the newborn and decreases notably from 20–21 days of age. A protocol that has proved effective is the three-stage strategy (IRT/DNA/IRT) being used by several screening programs [12]. The main problem in the determination of IRT is a higher than expected percentage of false positive tests in some communities. The hypothesis is that the reference range for IRT may vary depending upon the ethnicity of the newborn.

The newborns of families from the north of Africa have higher IRT values and most positive newborn screenings in this population could be considered “false positives” [13]. Higher IRT values have also been found to be associated with sick infants [14]. Despite these considerations, no special strategy for premature infants and sick newborns is recommended. However, it may be useful to record the ethnicity of the newborn to enable its association with possible deviations in the analytical results. Cutoff values at 48 hours of life can be set in different ways: an absolute value, typically 60–65 ng/ml, although values up to 90–105 ng/ml are acceptable, or a changing value based on the results obtained over a period of time, which may be a day or a month, accepting values higher than the 90th, 95th or 98th percentiles for that period as pathological. All these findings have been summarized in an excellent review by Therrell Jr et al. [15].

Moreover, these factors have been individually analyzed for their impact on IRT values. As a new feature of the findings of these authors, in this study we assessed the conditions (room temperature, gender, gestational age, age of the child at extraction, maternal age and ethnicity) that can alter the normal levels of IRT, after one year of experience using a three-stage strategy protocol (IRT/DNA/IRT) through multivariate analysis. Furthermore, we used an innovative approach to assess the influence of nutrition type on altered IRT values.

## Methods

### Study population

Newborns in the province of Alicante.

### Design and participants

Cross-sectional observational study. A sample of all newborns in the province of Alicante was selected to undergo neonatal screening (IRT/DNA/IRT) for metabolic diseases after informed consent was given by the parents or guardians (over 99% of the newborns) from March 2012 to February 2013. The dried blood sample on filter paper was required to meet the defined quality criteria [16]. Any child not meeting these requirements was excluded from the study. Additionally, all children with CF were excluded since IRT distribution data could be incorrect.

### Variables and measures

The primary variable was defined as altered levels of IRT. This alteration was considered to be an IRT value at or above 65 ng/ml. Quantification of IRT was performed using the AutoDELFIA Neonatal IRT kit from Perkin Elmer, initially establishing the cutoff value of IRT at 65 ng/ml, as per the experiences of the majority of Spanish laboratories performing this test according to the Spanish Neonatal Screening Association (AECNE) [17]. Secondary variables were: child’s gender, country of origin of the mother, maternal age (in years), gestational age (in weeks), type of feeding (natural, formula, mixed and special (nasogastric or intravenous), time from birth to blood collection and average temperature of the month of birth (in °C). The variables were grouped as follows: 1) Origin of the mother: Spain, Africa, America, Rest of Europe and Asia. This grouping was made based on the distribution of the origin of mothers in recent years carried out by the Department of Health of the Valencian Community [18]; 2) Maternal age: <20, 20–40 and >40 years of age. 3) Gestational age (in weeks): very preterm newborn <32, preterm from 32 to 37, and term ≥37. These intervals were chosen based on the recommendations of the Standards Committee of the Spanish Society of Neonatology [19]; and 4) day of extraction: <20 and ≥20. The cutoff was chosen according to the screening protocol based on quantification of IRT in a second sample after 20 days of life [17].

Quantification of IRT was performed using AutoDELFIA Neonatal IRT kit from Perkin Elmer and the average temperature of the month of birth was obtained from the Spanish Meteorological Agency (AEMET) [20]. The remaining variables were obtained from the neonatal screening report.

### Sample size

The final sample consisted of 13,310 children. With a 95% confidence level and a maximum expected proportion (p = q =0.5), the estimated error of the proportion of abnormal IRT was 0.85%.

### Statistical analysis

A descriptive analysis of the variables was performed. Absolute and relative frequencies were used for qualitative variables, while means, standard deviations and 95th and 99th percentiles were used for quantitative variables. Differences were analyzed using nonparametric tests between subgroups of gender, ethnicity, maternal age, gestational age, type of feeding and days from birth to extraction of the blood sample. A multivariate logistic regression model was implemented to estimate the adjusted odds ratios (ORs) with the aim of analyzing the relationship between atypical IRT and the study variables. The ORs were adjusted by gender, origin of the mother, maternal age, gestational age group, feeding group, day of extraction group and temperature. The goodness of fit of the model was performed using the likelihood ratio test. In addition, we worked with the predicted probabilities of atypical IRT from the multivariate model to create graphs to help interpret the results. All analyses were performed at the 5% significance level and for each relevant parameter its associated confidence interval (CI) was calculated. All analyses were performed using IBM SPSS Statistics 19.

### Missing data

The initial sample consisted of 14,877 newborns. However, those who did not have data for all the variables were excluded from this sample, leaving the sample size stated above.

### Ethical issues

Neonatal screening studies were approved by the Ethics Committee of the Valencian Community, requiring the informed consent of the newborn’s parent or guardian, in compliance with the current legislation in medical ethics. Moreover, the data, which are data from routine clinical practice in neonatal screening, were anonymized and encrypted, satisfying the data protection law.

## Results

The gestational age of the total sample was 39.0 (SD 1.9) weeks, age at extraction was 5.4 (SD 3.3) days and maternal age was 31.6 (SD 6.7) years. Average monthly temperatures in Alicante during the period studied ranged from a low in February of 12.1°C to a high of 27.7°C in August. IRT values were not distributed normally according to the Kolmogorov-Smirnov normality test (p <0.0001) with a mean of 22.2 (SD 13.7) ng/ml, 95th percentile of 45.8 ng/ml and 99th percentile of 69.4 ng/ml. Table 1 summarizes the IRT data according to the groups established for the different covariates in the total sample.

**Table 1:** **Immunoreactive trypsin values (in ng/ml) in newborn screening**

**Variable**	**Mean (SD)**	**95% CI**	**Percentile 95**	**Percentile 99**
*Gender:				
Male	21.5(13.7)	21.2-21.8	44.2	68.7
Female	22.8(13.5)	22.5-23.1	47.2	71.2
*Maternal origin:				
Spain	22.2(13.7)	21.9-22.4	45.6	69.3
Africa	24.0(17.2)	23.0-25.1	54.0	94.8
South America	20.8(11.3)	20.1-21.5	41.7	63.6
Rest of Europe	22.3(12.6)	21.5-23.0	45.6	71.6
Asia	20.1(12.1)	18.9-21.4	46.2	69.3
**Maternal age (y):				
<20	23.8(13.2)	22.5-25.1	52.0	73.3
20-40	22.1(13.7)	21.9-22.3	45.7	69.6
>40	22.9(14.2)	21.7-24.2	47.9	70.6
*Gestational age (w):				
<32	25.0(16.7)	23.2-26.8	53.9	83.0
32-37	23.2(11.7)	22.5-24.0	46.6	65.9
>37	22.0(13.8)	21.8-22.3	45.4	69.5
*Feeding methods:				
Natural	22.2(14.3)	21.9-22.4	46.1	71.5
Mixed	21.9(12.0)	21.4-22.4	44.0	62.8
Formula	22.2(12.4)	21.8-22.7	45.3	65.1
Special	53.3(52.9)	29.2-77.4	234.6	nd
*Extraction (d):				
<20	22.2(13.9)	22.0-22.5	46.1	70.3
≥20	15.4(19.8)	10.3-20.6	48.0	nd

*Kruskal-Wallis test (p < 0.001); **Kruskal-Wallis test (p < 0.01).

nd: not determined.

2012–2013 data for a Spanish region.

Table 2 summarizes the descriptive and analytical information from the study sample (n = 13,310) according to IRT cutoff values (65 ng/ml). Most of the mothers were between 20 and 40 years of age (94.1%) and Spanish (75.3%). Most newborns were at term (>37 weeks) (86.8%) and were breastfeeding (65.0%).

**Table 2:** **Analysis of atypical immunoreactive trypsin (≥65 ng/ml) in newborns in a Spanish region**

**Variable**	**Total 13310 n(%)/x ± s**	**AIT 196 (1.5%) n(%)/x ± s**	**Not AIT 13114 (98.5%) n(%)/x ± s**	**Adj. OR**	**95% CI (Adj. OR)**	**p-value**
Gender male	6861(51.5)	95(48.5)	6766(51.6)	0.88	0.66-1.17	0.385
Maternal origin:						
Spain	9994(75.1)	144(73.5)	9850(75.1)	1	1	0.219
Africa	953(7.2)	22(11.2)	931(7.1)	1.50	0.95-2.37	
South America	967(7.3)	9(4.6)	958(7.3)	0.63	0.32-1.25	
Rest of Europe	1061(8.0)	15(7.7)	1046(8.0)	0.88	0.51-1.52	
Asia	335(2.5)	6(3.1)	329(2.5)	1.21	0.52-2.82	
Maternal age (years):						
<20	358(2.7)	8(4.1)	350(2.7)	1.70	0.83-3.49	0.353
20-40	12531(94.1)	182(92.9)	12349(94.2)	1	1	
>40	421(3.2)	6(3.1)	415(3.2)	1.06	0.46-2.40	
Gestational age (weeks):						
<32	146(1.1)	6(3.1)	140(1.1)	1.45	0.49-4.25	0.137
32-37	1568(11.8)	13(6.6)	1555(11.9)	0.59	0.33-1.05	
>37	11596(87.1)	177(90.3)	11419(87.1)	1	1	
Feeding method:						
Natural	8698(65.3)	144(73.5)	8554(65.2)	1	1	<0.001
Mixed	1902(14.3)	16(8.2)	1886(14.4)	0.53	0.31-0.89	
Formula	2689(20.2)	30(15.3)	2659(20.3)	0.72	0.48-1.07	
Special	21(0.2)	6(3.1)	15(0.1)	21.88	6.92-69.14	
Days from extraction >20	47(0.4)	1(0.5)	46(0.4)	1.51	0.21-11.13	0.684
Temperature (°C)	18.7 ± 5.6	18.0 ± 5.2	18.8 ± 5.6	0.98	0.95-1.00	0.064

*Abbreviations*: *AIT* atypical immunoreactive trypsin, *Adj. OR* adjusted odds ratio, *CI* confidence interval.

Goodness-of-fit of the model: *Χ* ^2^ = 53.19, p < 0.001.

ORs were adjusted for: gender, maternal origin, maternal age, gestational age, feeding method, days from extraction and temperature.

2012–2013 data.

The magnitude of atypical IRT was 1.5% (95% CI: 1.27-1.68%). Associated factors: male (OR = 0.88, 95% CI: 0.66-1.17, p = 0.385), maternal origin (Spain → OR = 1; Africa → OR = 1.5, 95% CI: 0.95-2.37; South America → 0.63, 95% CI: 0.32-1.25; rest of Europe → 0.88, 95% CI: 0.51-1.52; Asia → 1.21, 95% CI: 0.52-2.82; p = 0.219), maternal age (<20 → OR = 1.70, 95% CI: 0.83-3.49; 20-40 → OR = 1; >40 → OR = 1.06, 95% CI: 0.46-2.40; p = 0.353), gestational age (<32 → OR = 1.45, 95% CI: 0.49-4.25; 32-37 → OR = 0.59, 95% CI: 0.33-1.05; >37 → OR = 1; p = 0.137), feeding method (natural → OR = 1; mixed → OR = 0.53, 95% CI: 0.31-0.89; formula → OR = 0.72, 95% CI: 0.48-1.07; special → OR = 21.88, 95% CI: 6.92-69.14; p < 0.001), >20 days from extraction (OR = 1.51, 95% CI: 0.21-11.13, p = 0.684), and temperature (OR = 0.98, 95% CI: 0.95-1.00, p = 0.064).

**Figure 1:**
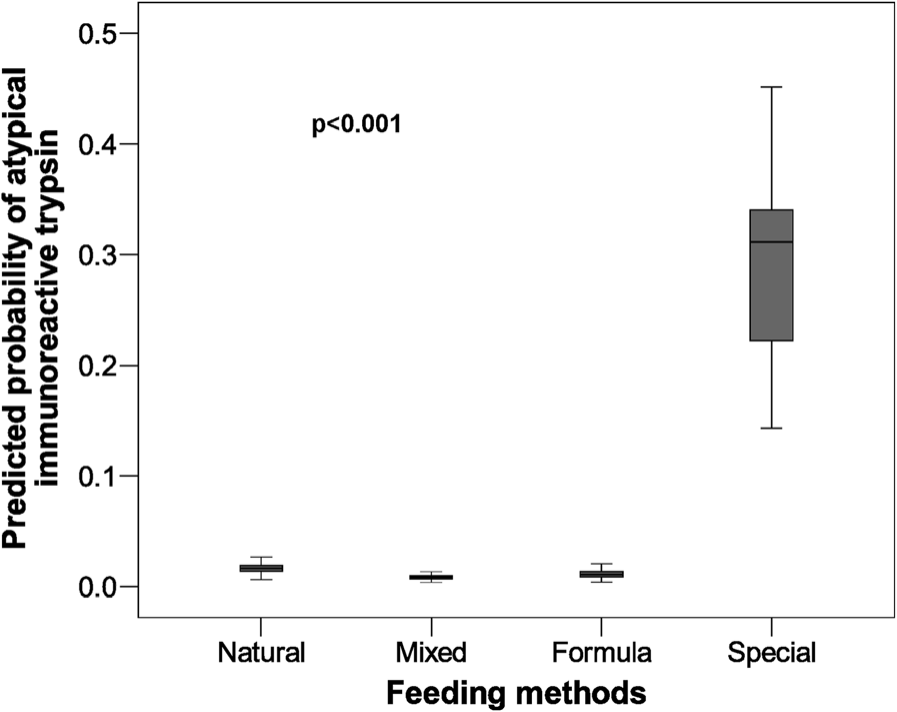
**Predicted probability of atypical immunoreactive trypsin between feeding methods in newborns in a Spanish region.** 2012–2013 data.

On a Cartesian graph (Figure 1) feeding groups were plotted on the X-axis and predicted probabilities of atypical IRT on the Y-axis. This graph shows that the newborns who received special nutrition were more likely to have atypical IRT.

## Discussion

When the differences in the IRT concentration in the total sample were studied according to type of feeding, IRT concentrations did not vary significantly between the three feeding groups (breastfeeding, formula and mixed). However, the elevated IRT levels in neonates receiving special feeding was striking, showing a significant difference. The mean IRT rose to more than double that of the other newborns, with many more of these newborns having IRT values above the cutoff. This was confirmed in the multivariate analysis, which showed that the probabilities of newborns receiving special nutrition being above the cutoff were about 20 times higher than those who were fed. This is undoubtedly associated with the fact that these children were subjected to special nutrition, due to their inherent disease, causing the increase in IRT. Although IRT is elevated in individuals with CF, an increase in IRT has also been seen in normal individuals with an immature ductal system, carriers of CF (66%), and in neonates with other diseases such as trisomy of chromosome 13, 18 and 21 [21], congenital infections (cytomegalovirus and other subclinical infections), renal failure, inadequate pancreatic perfusion and intestinal atresia. Higher IRT values are also associated with perinatal asphyxia [22] and sick infants [14]. On the other hand it has been reported that prenatal stress may be responsible for up to 25% of positive cases [22].

When IRT concentrations were examined by maternal ethnicity, clearly higher values were observed in newborns of African origin, with significant differences, a finding consistent with the literature [23]. In addition, slightly lower IRT values were found in infants of Asian and American mothers. On comparing newborns of African ethnicity with the rest of the sample using multivariate analysis, there was an almost significant difference and a greater probability (OR 1.6) that the neonate of an African mother was more likely to have IRT levels above the reference value. Ethnic influence on IRT level has been studied previously [24], showing an overrepresentation of African Americans in screen-positive newborn infants (10% of the general population, rising to 28% in the positive population) despite the lower incidence of CF in this population. Note that in the present study maternal origin was divided into: Spain, rest of Europe, Africa, America and Asia; although Africa here corresponded primarily to countries in north Africa (mainly Morocco). Thus, newborns from north African families have higher IRT values and most of the positive newborn screens in this population could be considered “false positives” [13]. It should be noted that CF is the most common genetic disorder among Caucasian children. The incidence is variable: it is much less common in Asian and African populations than in European and North American populations, with variations within each country. The prevalence varies between a maximum of 1/2000 in Ireland and a minimum of 1/500,000 in Japan [23,24].

With respect to the other factors analyzed in this study, most behaved similarly to findings previously described by other authors [15,23,24], with the exception of temperature. This is possibly due to the variability between minimum and maximum temperatures throughout the year being much lower than in the regions in which this association has been observed [25]. Finally, there was no association with maternal age. This must be verified by future studies.

This work shows the need to establish cutoffs in IRT values adjusted to the ethnic and prematurity characteristics of the study population, as well as the need to postpone screening in those infants with conditions that require special feeding. This has the potential to reduce the number of inconclusive results, in which a second marker must be measured, either through DNA mutation analysis or taking a second sample for IRT retesting, thereby resulting in lower economic and emotional costs to the parents caused by unnecessary confirmations.

### Strengths and limitations

The main strength of this study is its innovative approach to analyzing nutrition type when altered IRT values are present. Ethnicity is also evaluated in a manner different from that used by other authors. Furthermore, these factors were analyzed taking into account the ratio of all the altered IRT values, that is, through a mathematical multivariate model. Finally, the random error was less than 1%. As limitations, the IRT values analyzed are only useful for the population studied. Each neonatal cystic fibrosis screening center must define its own cutoff values and to do this the same methodology used in this work could be followed. Moreover, to minimize information bias, great care was taken in the collection of all variables. In addition, to avoid selection bias, all children who underwent neonatal screening were included. Finally, although the statistical significance of the special type of nutrition was quite high, a wide confidence interval was obtained, therefore, in order to quantify OR more precisely, future studies using a larger sample size are needed.

## Conclusions

In conclusion, newborns receiving special nutrition have much higher IRT values, with a 20-times greater likelihood of being above the set reference value, no doubt due to the underlying disease state. Thus, CF screening is advisable in sick infants with a special diet once breastfeeding, formula or mixed feeding is initiated. Newborns of African ethnicity, specifically children born to north African mothers, have higher IRT levels than those of other ethnic groups. An important factor to bear in mind is the increased ethnic variability resulting from increased migration.

The main learning point from this study is that we have to adopt new IRT cutoff points for children of certain ethnic backgrounds and for those who follow a special diet. However, we must be cautious, as these results must be verified by other authors in studies incorporating a large number of newborns.

## Abbreviations

CF: Cystic fibrosis
IRT: Immunoreactive tryspin
DNA: Deoxyribonucleic acid
AECNE: *Asociación Española de Cribado Neonatal* [Spanish Neonatal Screening Association]
OR: Adjusted odds ratio
